# Supplementary material for: In-situ monitoring of interface proximity effects in ultrathin ferroelectrics
Source: Nat Commun. 2020 Nov 16;11:5815. doi: 10.1038/s41467-020-19635-7 (PMC7669862; doi:10.1038/s41467-020-19635-7)
Supplement: Supplementary file 1 — Supplementary Information [file 41467_2020_19635_MOESM1_ESM.pdf]

Supplementary information

In-situ monitoring of interface proximity effects in ultrathin ferroelectrics

Nives Strkalj[1,†,*], Chiara Gattinoni[1], Alexander Vogel[2,†], Marco Campanini[2], Rea Haerdi[1], Antonella Rossi[1,3], Marta D. Rossell[2], Nicola A. Spaldin[1], Manfred Fiebig[1] and Morgan Trassin[1,*]

[1]*Department of Materials, ETH Zürich, 8093 Zürich, Switzerland.*

[2]*Electron Microscopy Center, Swiss Federal Laboratories for Materials Science and Technology, Empa, 8600 Dübendorf, Switzerland.*

[3]*Department of Chemical and Geological Sciences, University of Cagliari, 09124 Cagliari, Italy*

This file contains Supplementary Notes 1–4.

1 Details of the growth conditions, termination control, thickness and ISHG monitoring

The thin films and heterostructures were grown on TiO_2-terminated (001) STO (vicinal angle $<0.1°$) substrates without pretreatment (Crystec GmbH). Layer-by-layer growth mode enables us to engineer the top termination of PTO films. Starting from the substrate, the perovskite ABO_3 termination of the top interface is set to the BO_2 plane. By buffering the STO with 2 u. c. of SRO (001), Ru volatility reverses the termination of the top interface to the AO plane. The set termination as BO_2 plane or AO is then kept for the subsequent LSMO and PTO layers.

To compensate the bound charge at the bottom interface, 15 u. c. of LSMO (001) were deposited as the bottom electrode. Uniaxial ferroelectric PTO (001) films with a thickness of 15–25 u. c. were grown with polarization set upwards on STO||SRO|LSMO heterostructure and polarization set downwards on STO||LSMO|PTO heterostructure. In Supplementary Fig. 1a,b, the time-dependent RHEED intensity is shown for growths of PTO and LSMO layers. The ISHG data of the entire PTO growth process for both interface configurations are shown in Supplementary Fig. 1c,d. The final ISHG intensity of the competitive interfaces is four times lower than the final ISHG intensity of the cooperative interfaces.

We note that the growth environment during deposition (partial oxygen pressure, UV irradiation from the PLD plume, and plasma exposition) may also have an impact on the final polarization state. Previous work highlighted the charge screening of the oxygen-rich environment during the PLD growth of $BaTiO_3$ thin films[1]. Here, the influence of the PLD plume was ruled out for the following reason. The front of the plume is rich in heavier ions, in our case cations, and is therefore

positively charged[2]. Such a positively charged environment would preferably screen negatively charged surfaces (downwards polarized) during the growth and create a suppression of polarization once the growth is interrupted. Our observations, in contrast, reveal a delayed onset of the polarization and the polarization enhancement once the growth is stopped for the downwards-polarized films. UV radiation caused by the plasma plume could further affect the surface chemistry[3]. UV-enhanced reactivity at the top interface could contribute to the observed recovery of the polarization state once the growth is resumed.

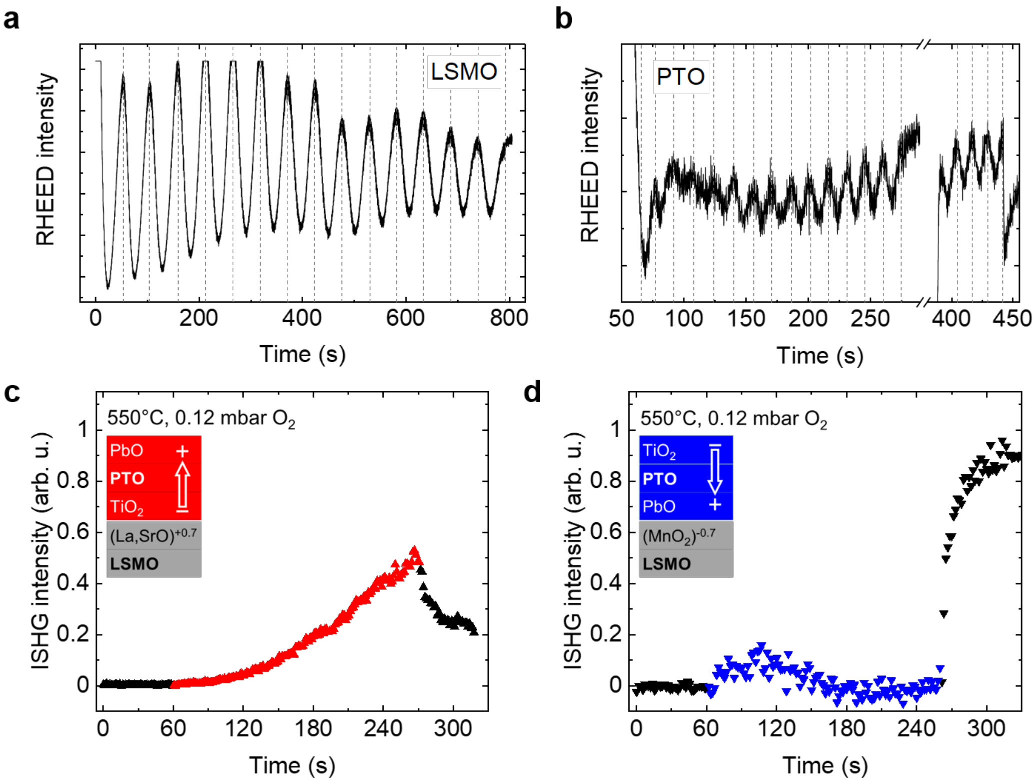

Supplementary Figure 1: **RHEED monitoring of thickness during growth of LSMO and PTO films and ISHG monitoring of polarization during growth of PTO films. a**, **b**, Time-dependent RHEED monitoring of the LSMO and PTO layers in the STO|LSMO|PTO heterostructure. **c**, **d**, ISHG signal during growth (filled red and blue symbols) and during growth interruptions (filled black symbols) for configurations of (c) competitive and (d) cooperative interfaces of PTO films with 20 u. c. thickness. The inset shows the chemistries of interfacial planes at the PTO|LSMO interface and the polarization direction set by the bottom interface.

2 ISHG determination of critical thickness and first-principle calculations of the band alignment at the PTO|LSMO interface.

Experimentally, a higher critical thickness was observed for the MnO_2|PbO-terminated bottom interface (downwards-polarized sample) than the $La_{0.7}Sr_{0.3}O$|TiO_2-terminated bottom interface (upwards-polarized sample), see Supplementary Fig. 2a,b.

We compare the two LSMO|PTO interfaces with MnO_2|PbO and $La_{0.7}Sr_{0.3}O$|TiO_2 terminations, using density of states (DOS) calculations, see Supplementary Fig. 2c,d. The structure of PTO is set to paraelectric and no relaxations were allowed in the system. The Schottky barrier for electrons and holes is ϕ_n=$E_C - E_F$ and ϕ_p=$E_F - E_V$, respectively, where E_C, E_V and E_F are the conduction-band minimum and valence-band maximum away from the interface, and the Fermi energy, respectively. For the MnO_2|PbO termination, where the polarization is set upwards, the Schottky barrier for electrons, ϕ_n, is ~ 0.1 eV. For the $La_{0.7}Sr_{0.3}O$|TiO_2 termination, where the polarization is set downwards, the Schottky barrier for holes, ϕ_e, is ~ 0.7 eV. Since the potential barrier for electronic reconstructions is greater in $La_{0.7}Sr_{0.3}O$|TiO_2 than in MnO_2|PbO, a higher critical thickness is expected.

Absence of critical thickness in Pb-based ferroelectrics has been reported theoretically and experimentally. In theoretical studies[4,5], this has been observed in heterostructures with metal screening at both interfaces. In experimental studies on thin films[6,7], the ionic adsorbates from the environment or internal defects were suggested as the screening mechanism. We thus conclude that, similarly to the above and in line with the findings in Ref. [8] on PTO films on SRO, the

stabilization of ultrathin ferroelectricity for our PTO film can be attributed to interface effects[7].

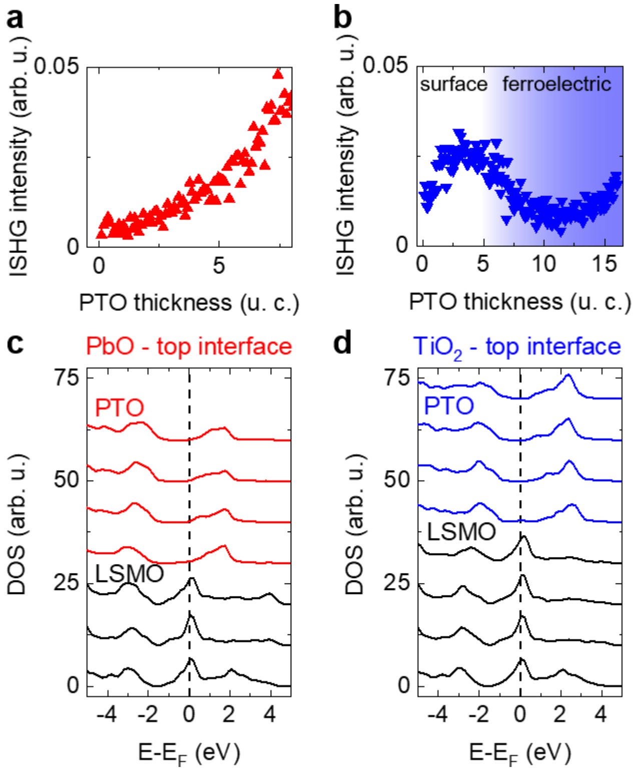

Supplementary Figure 2: **ISHG monitoring during the early stage of growth of PTO films and the first-principle calculation of the band alignment at the PTO|LSMO interface.** **a**, **b**, The ISHG data of the early stage of the PTO growth for polarization set upwards and downwards from the bottom interface in red and blue, respectively. **c**, **d**, DOS calculations for the LSMO|PTO interface with PbO and TiO_2 top interface terminations.

3 Reproducibility and generality

To demonstrate the reproducibility of our observations, we show the time-dependent evolution of the ISHG signal during growth of several PTO samples with competitive and cooperative interfaces in Supplementary Fig. 3a,b. The deposition rate is on average 4.8 u. c. per min, the same as in Supplementary Note 1.

We further monitored the in-situ polarization dynamics by ISHG for: BTO (Supplementary Fig. 4a), PZT (Supplementary Fig. 4b,c) and BFO (Supplementary Fig. 4d,e). For BTO, we observe no polarization dynamics during the growth interruption, while for A-site volatile PZT and BFO, we observe polarization dynamics equivalent to the one of PTO: suppression for upwards-polarized layers and enhancement for downwards-polarized layers during the growth interruption.

We examined the influence of other types of adatoms in ab-initio calculations. We present the effects of a hydrogen adatom on the polarization in PTO films in Supplementary Fig. 5a,b which are qualitatively equivalent to the effects obtained in the case of a Pb adatom presented in Fig. 3.

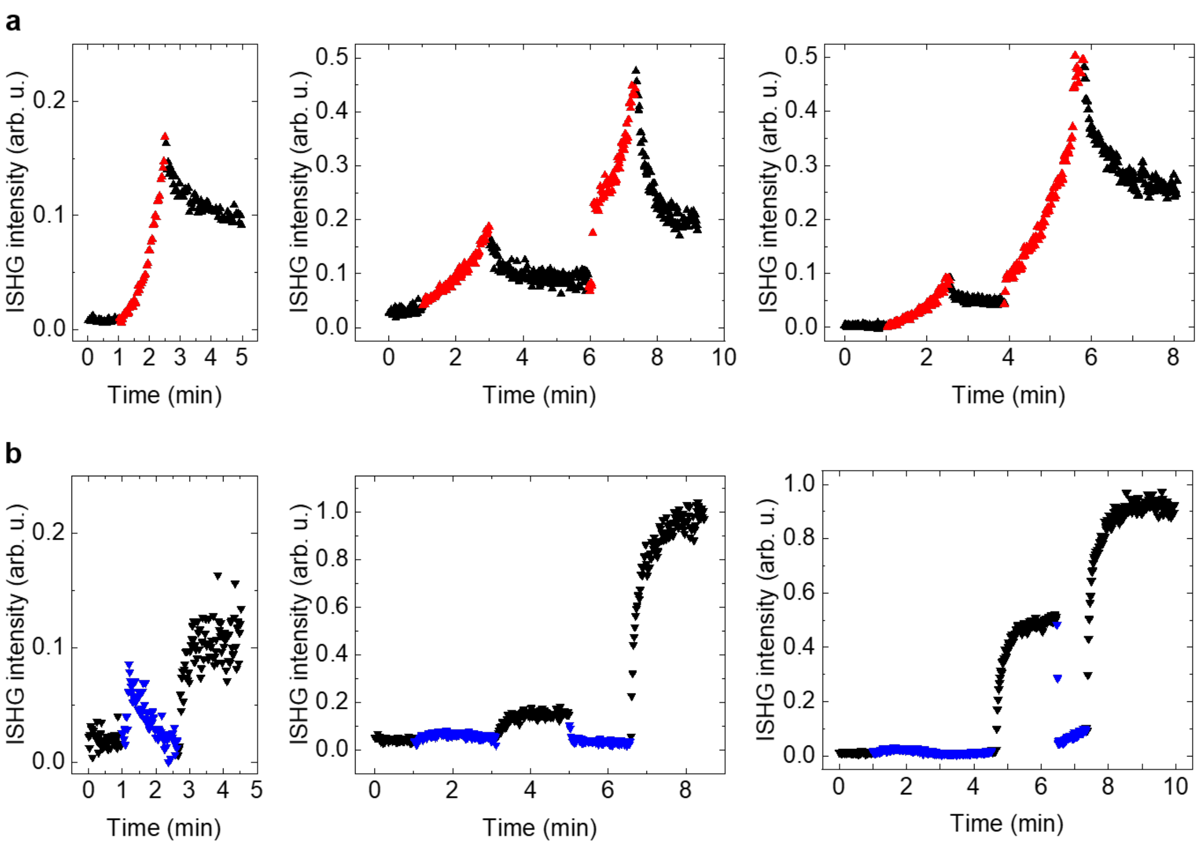

Supplementary Figure 3: **Reproducibility of ISHG monitoring of polarization during growth of PTO films.** **a**, **b**, ISHG signal probing the thin-film polarization during growth (filled red and blue symbols) and during growth interruptions (filled black symbols) for PTO layers with (a) competitive and (b) cooperative interfaces.

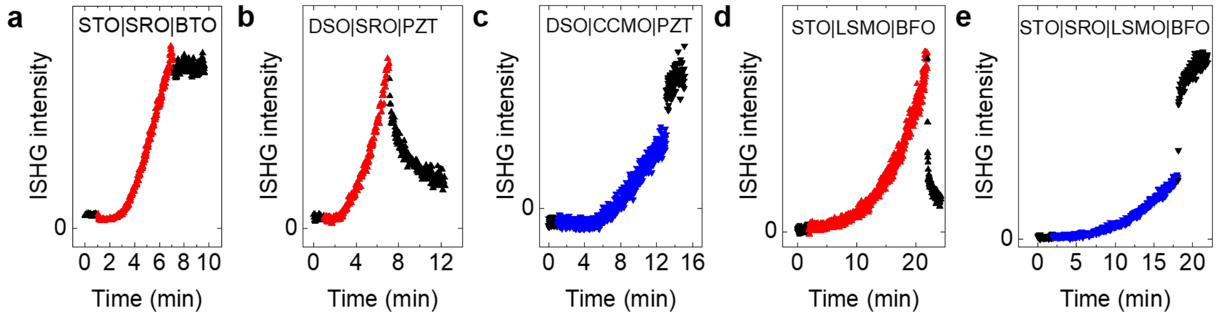

Supplementary Figure 4: **ISHG monitoring of polarization during growth of BTO, PZT and BFO films.** **a–e**, ISHG signal of the thin-film polarization during growth (filled red and blue symbols) and during growth interruptions (filled black symbols) for (a) a BTO film of 30 u. c. thickness on an SRO-buffered STO, (b) a PZT film of 25 u. c. thickness on an SRO-buffered (110)-oriented $DyScO_3$ (DSO), (c) a PZT film of 60 u. c. thickness on a $Ca_{0.96}Ce_{0.04}MnO_3$ (CCMO)-buffered DSO, (d) a BFO film of 40 u. c. thickness on a LSMO-buffered STO, and (e) a BFO film of 30 u. c. thickness on an SRO- and LSMO-buffered STO. Red and blue denote the direction of polarization set upwards and downwards from the bottom interface, respectively.

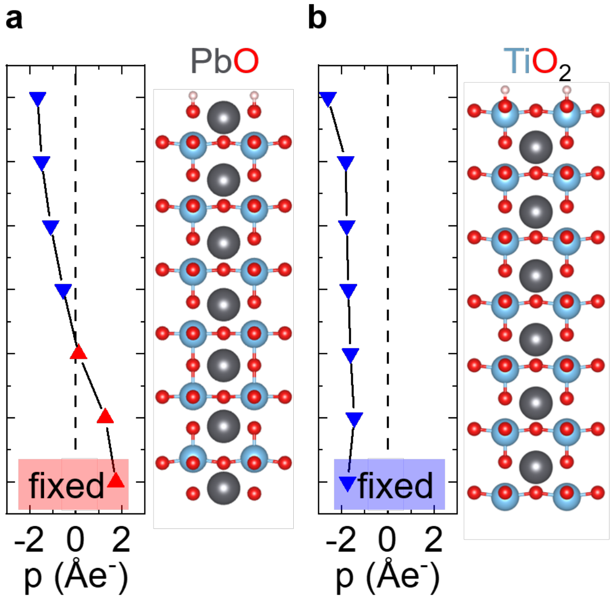

Supplementary Figure 5: **Density functional calculations of dipole moments in PTO with a H adatom per surface u. c.** **a**, **b**, Dipole moments (p) perpendicular to the bottom interface. The polarization direction is fixed at the bottom interface to match with our experiments (marked as "fixed"). The atomic positions in heterostructures with (a) PbO and (b) TiO_2 top-interface termination and their ionic positions are depicted on the right hand side of the graphs.

4 STEM tetragonality and composition analysis of the PTO films, and polarization mapping in the PTO|STO heterostructure

The tetragonality within the bulk of the PTO layer, excluding the 3 u. c. next to interfaces, is about 1.06 in both PTO films with competitive and cooperative interfaces[9], see Supplementary Fig. 6a,b. Composition analysis of the PTO top interface obtained by energy-dispersive X-ray (EDX) spectroscopy in STEM mode is shown in Supplementary Fig. 7a,b. We find an amorphous Pb-rich layer at the top interface in both interface configurations. In contrast to the cooperative configuration, post-deposition STEM mapping of dipole moments reveals the absence of net polarization in the STO|PTO heterostructure for the PTO layer with competitive interfaces, see Supplementary Fig. 8.

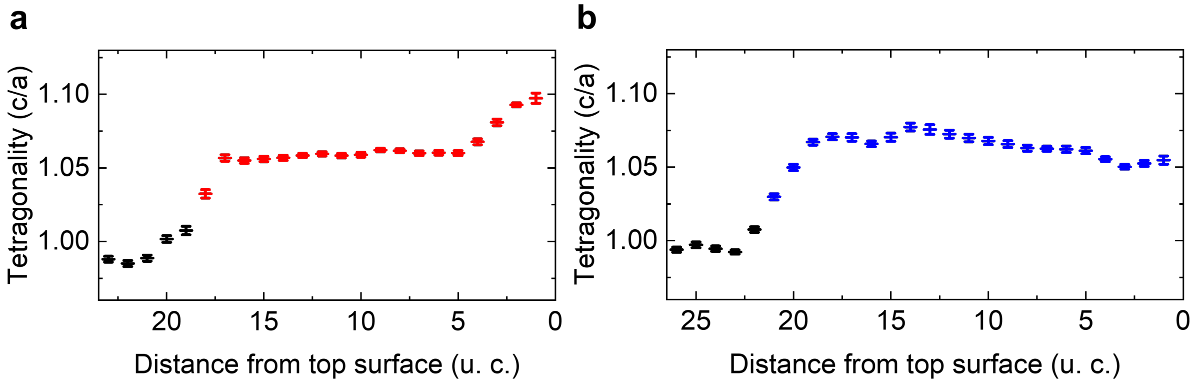

Supplementary Figure 6: **STEM tetragonality a**, **b**, Tetragonality for PTO films with (a) competitive (red) and (b) cooperative (blue) interfaces. The error bars are the standard error of the mean.

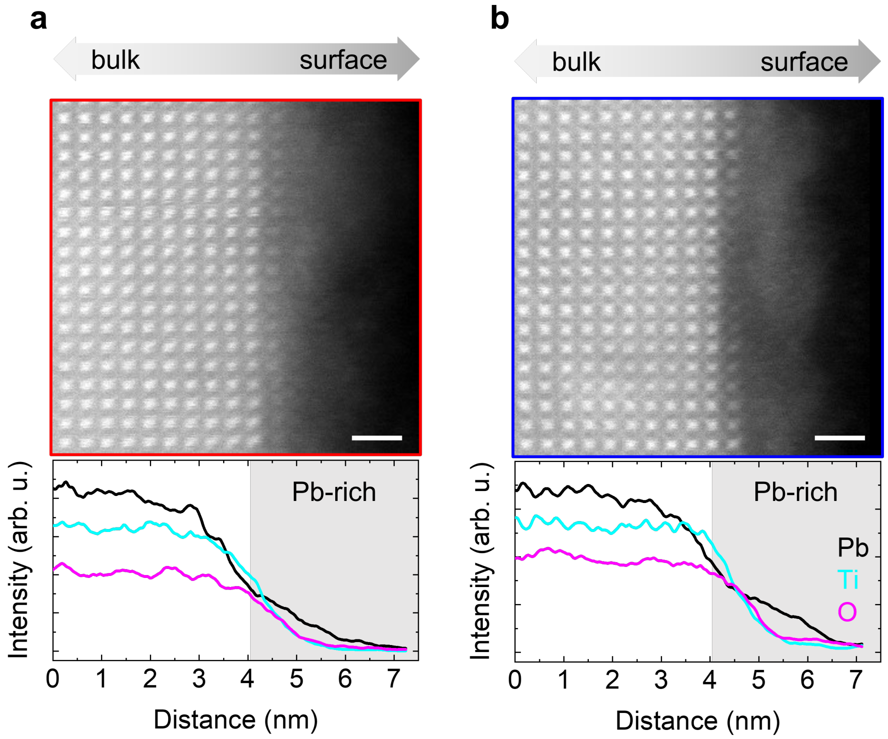

Supplementary Figure 7: **EDX composition analysis of the PTO top interface region**, **a**, **b**, Atomic resolution HAADF-STEM images of the top interface of the PTO film of 20 u. c. thickness (top). Corresponding elemental line profiles over a scanning distance of 7 nm for Pb, Ti and O for PTO films with (a) competitive and (b) cooperative interfaces (bottom) from the center of the PTO layers (0 nm) towards the surface. The EDX elemental line profiles of Pb, Ti and O were calculated from the Pb-L, Ti-K, and O-K signals of the EDX spectrum image. Scale bars are 1 nm.

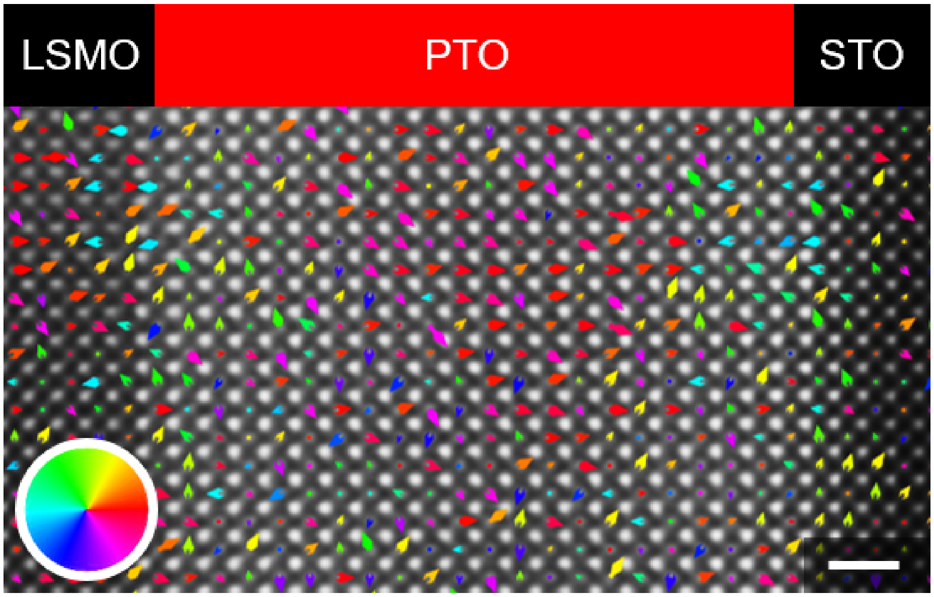

Supplementary Figure 8: **Absence of the PTO net polarization in a STO|PTO heterostructure.** Post-deposition STEM mapping of PTO dipole moments after STO capping of the PTO layer with competitive interfaces. The arrows show the direction (color wheel) and amplitude (arrow length) of the dipole moments. Scale bar is 1 nm.

1. Strkalj, N., De Luca, G., Campanini, M., Pal, S., Schaab, J., Gattinoni, C., Spaldin, N. A., Rossell, M. D., Fiebig, M. & Trassin, M. Depolarizing Field Effects in Epitaxial Capacitor Heterostructures. *Phys. Rev. Lett.* **123**, 147601 (2019).

2. Ojeda-G-P, A., Döbeli, M. & Lippert, T. Influence of Plume Properties on Thin Film Composition in Pulsed Laser Deposition. *Adv. Mater. Interfaces* **5**, 1701062 (2019).

3. Hensling, F. V. E., Keeble, D. J., Zhu, J., Brose, S., Xu, C., Gunkel, F., Danylyuk, S., Nonnenmann, S. S., Egger, W. & Dittmann, R. UV radiation enhanced oxygen vacancy formation caused by the PLD plasma plume. *Sci. Rep.* **8**, 8846 (2018).

4. Stengel, M., Vanderbilt, D. & Spaldin, N. A. Enhancement of ferroelectricity at metal–oxide interfaces. *Nat. Mater.* **8**, 392–397 (2009).

5. Sai, N., Kolpak, A. M. & Rappe, A. M. Ferroelectricity in Ultrathin Perovskite Films. *Phys. Rev. B* **72**, 020101 (2005).

6. Fong, D. D., Kolpak, A. M., Eastman, J. A., Streiffer, S. K., Fuoss, P. H., Stephenson, G. B., Thompson, C., Kim, D. M., Choi, K. J., Eom, C. B., Grinberg, I. & Rappe, A. M. Stabilization of Monodomain Polarization in Ultrathin $PbTiO_3$ Films. *Phys. Rev. Lett.* **96**, 127601 (2006).

7. Gao, P., Zhang, Z., Li, M., Ishikawa, R., Feng, B., Liu, H.-J., Huang, Y.-L., Shibata, N., Ma, X., Chen, S., Zhang, J., Liu, K., Wang, E.-G., Yu, D., Liao, L., Chu, Y.-H. & Ikuhara, Y. Possible absence of critical thickness and size effect in ultrathin perovskite ferroelectric films. *Nat. Commun.* **8**, 15549 (2017).

8. Gattinoni, C., Strkalj, N., Härdi, R., Fiebig, M., Trassin, M. & Spaldin, N. A. Interface and surface stabilization of the polarization in ferroelectric thin films. *accepted* (2020).

9. Jia, C.-L., Nagarajan, V., He, J.-Q., Houben, L., Zhao, T., Ramesh, R., Urban, K. & Waser, R. Unit-cell scale mapping of ferroelectricity and tetragonality in epitaxial ultrathin ferroelectric films. *Nat. Mater.* **6**, 64–69 (2007).
